# Velocity-Free Attitude Control of Quadrotors: A Nonlinear Negative Imaginary Approach

**DOI:** 10.3390/s21072387

**Published:** 2021-03-30

**Authors:** Ahmed G. Ghallab, Ian R. Petersen

**Affiliations:** Research School of Engineering, The Australian National University, Canberra, ACT 2601, Australia; ahmed.ibrahim@anu.edu.au

**Keywords:** nonlinear negative imaginary systems, quadrotors, attitude control, robustness, feedback stability

## Abstract

In this paper, we propose a new approach to the attitude control of quadrotors, by which angular velocity measurements or a model-based observer reconstructing the angular velocity are not needed. The proposed approach is based on recent stability results obtained for nonlinear negative imaginary systems. In specific, through an inner-outer loop method, we establish the nonlinear negative imaginary property of the quadrotor rotational subsystem. Then, a strictly negative imaginary controller is synthesized using the nonlinear negative imaginary results. This guarantees the robust asymptotic stability of the attitude of the quadrotor in the face of modeling uncertainties and external disturbances. First simulation results underline the effectiveness of the proposed attitude control approach are presented.

## 1. Introduction

In recent decades, unmanned aerial vehicles (UAVs) have seen increasing interest within the research communities and industry due to their potential for numerous applications including, for instance, inspection, surveillance, data acquisition, and military applications. Their potential future applications include search and rescue, border patrol, surveillance of wildfires, surveillance of traffic and land surveys. An important type of UAVs are quadrotors, which have useful properties such as a simple structure, and low operation and manufacturing costs [1,2]. Numerous control methods have been proposed in order to tackle the quadrotor stability problem, see, e.g., [3,4,5,6].

In many of the aforementioned applications of quadrotors, designing an attitude controller with high level of performance and reliability is crucial. In the existing literature, a typical method of estimating the quadrotor’s attitude assumes that the angular velocity measurements are required. However, the velocity measurements in quadrotors can be noisy or even not available. Furthermore, using observer-based methods to reconstruct the angular velocity normally lead to inaccurate estimation of the velocity and, hence, degrade the attitude control performance [7,8,9,10]. This suggests using direct measurements of the attitude rather than indirect estimations.

Theoretically, different methods can be used to measure the attitude of the quadrotor such as: Euler angles, direction cosine matrices, and quaternions. However, the Euler angles have the advantage of being more physically sensible and easier to use, as each of the three Euler angles represents elementary rotations around the three principles axes of the quadrotor: roll, pitch, and yaw. Euler angles can be determined directly based on measurements earth’s magnetic field on the three body axes of the quadrotor. Sensors like AMR (anisotropic magneto resistive) magnetometers [11] are used to measure earth’s magnetic field.

Motivated by the forgoing, we aim in this paper to design an attitude control scheme for quadrotor systems where the angular velocity measurements or a model-based observer reconstructing the angular velocity are not required. The proposed approach rely on using the nonlinear negative imaginary systems framework, which is recently developed in [12] for nonlinear systems which are passive from the input to the derivative of the output (rather than the output as in the classical passivity theory). The nonlinear negative imaginary property of the quadrotor system will be established, and we shall employ the stability robustness results of [12] to design a velocity-free attitude controller by direct use of the Euler angles. For future work, the nonlinear negative imaginary approach can be employed or combined with other techniques as in [13,14,15] to provide the full flight control to quadrotor systems.

The rest of the paper is organized as follows: In Section 2, main related definitions and robust stability results from the linear/nonlinear negative imaginary literature are reviewed. In Section 3, we use the Euler–Lagrange dynamics of quadrotors systems to establish the nonlinear negative imaginary property of these systems. We use in Section 4, an inner-outer loop technique to design a velocity-free stabilizing controller for the attitude of quadrotors based on recently obtained nonlinear NI stability results. Finally, in Section 5, simulation results and some concluding remarks along with future directions are provided.

## 2. Preliminaries

Negative imaginary (NI) systems theory has been introduced in [16] for the control of flexible structures with colocated force actuators and position sensors. NI systems theory has seen significant progress in theory and application in the last decade, see for instance [17,18]. In this section, we review some of the related definitions and stability results from the NI literature in both the linear and nonlinear case.

### 2.1. Negative Imaginary Systems: Linear Case

We consider here the following linear time invariant (LTI) system:

(1) $$\begin{array}{cc} \; & \dot{x}\left(t\right)=Ax\left(t\right)+Bu\left(t\right), \end{array}$$

(2) $$\begin{array}{cc} & y(t)=Cx(t)+Du(t) \end{array}$$

where the matrices $A\in R^{n\times n},B\in R^{n\times m}$, $C\in R^{m\times n}$, and $D\in R^{m\times m}$. Assume that the system (1) and (2) has the m×m real-rational proper transfer function $G\left(s\right):=C{\left(sI-A\right)}^{-1}B+D$. The frequency domain characterization of the NI property of the above LTI system is given in the following definition.

**Definition** **1** ([19])**.** *A square transfer function matrix G(s) is called negative imaginary if the following conditions are satisfied:*

*1.*  G(s) has no pole at the origin and in ℜ[s]>0;
*2.*  For all ω>0, such that jω is not a pole of G(s), and $j\left(G\left(j\omega \right)-G{\left(j\omega \right)}^{T}\right)\geq 0$;
*3.*  If $j\omega_{0}$; $\omega_{0}\in \left(0,\infty \right)$, is a pole of G(jω), it is at most a simple pole and the residue matrix $K_{0}=\lim_{s\to j\omega_{0}}\left(s-j\omega_{0}\right)sG\left(s\right)$ is positive semidefinite Hermitian.

A linear time invariant system of the form (1) and (2) is NI if its transfer function is NI. An equivalent time-domain definition of the NI property for the LTI system (1) and (2) is given in the following lemma.

**Lemma** **1.** *Suppose that the system *(1)* and *(2)* (with D=0) is controllable and observable. Then, G(s) is negative imaginary if and only if there exists matrix P as in LMI *(4)* such that along the trajectories of the system, the function $V\left(x\right)=\frac{1}{2}x^{T}Px$ satisfies*

(3) $$\dot{V}\left(x\left(t\right)\right)\leq \dot{y}\left(t\right)u\left(t\right),\;\forall \;t\geq 0.$$

A strict notion of the negative imaginary property of the above LTI system is provided in the following definition.

**Definition** **2** ([19])**.** *A square transfer function matrix G(s) is strictly negative imaginary (SNI) if:*

*1.*  G(s) has no poles in ℜ[s]≥0;
*2.*  $j[G\left(j\omega \right)-G^{T}\left(j\omega \right)]>0$ for ω∈(0,∞).

The next two lemmas provide a state-space characterization of the NI and SNI properties for the LTI system (1) and (2), respectively.

**Lemma** **2** ([20])**.** *Let (A,B,C,D) be a minimal state-space realization of the transfer function matrix G(s). Then G(s) is negative imaginary if and only if det(A)≠0, $D=D^{T}$ and there exist matrices $P=P^{T}>0$, $W\in R^{m\times m}$, and $L\in R^{m\times n}$ such that the following LMI is satisfied:*

(4) $$\left[\begin{array}{cc} PA+A^{T}P & PB-A^{T}C^{T}\\ B^{T}P-CA & -(CB+B^{T}C^{T}) \end{array}\right]=\left[\begin{array}{cc} -L^{T}L & -L^{T}W\\ -W^{T}L & -W^{T}W \end{array}\right]\leq 0.$$

**Lemma** **3** ([21])**.** *Let (A,B,C,D) be a minimal state-space realization of the transfer function matrix G(s). Then G(s) is strictly negative imaginary if and only if:*

*1.*  det(A)≠0, $D=D^{T}$;
*2.*  *there exists a matrix $P=P^{T}>0,P\in R^{n\times n}$, such that*
  $$AP^{-1}+P^{-1}A^{T}\leq 0,\;\mathrm{and}\;\;B+AP^{-1}C^{T}=0;$$
*3.*  the transfer function matrix $M\left(s\right)∽\left[\begin{array}{c} \begin{array}{cc} A & B\\ LPA^{-1} & 0 \end{array} \end{array}\right]$ has full column rank at s=jw for any ω∈(0,∞) where $L^{T}L=-AP^{-1}-P^{-1}A^{T}$. That is, rank M(jω)=m for any ω∈(0,∞).

**Figure 1:**
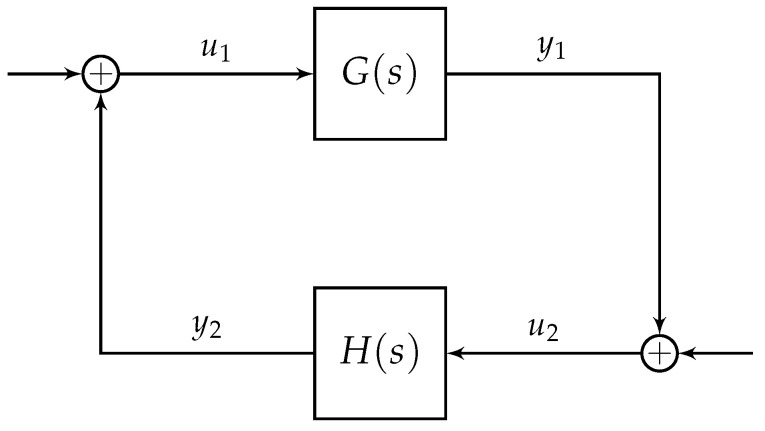
Positive feedback control systems of NI systems.

The stability robustness of a positive feedback interconnection of NI system is established in the following theorem:

**Theorem** **1.** *[16] Assume G(s) is a negative imaginary system with no poles at the origin and H(s) is a strictly negative imaginary system such that G(∞)H(∞)=0 and H(∞)≥0. Then, the positive feedback interconnection of G(s) and H(s), as in Figure 1, is internally stable if and only if*

$$\lambda_{max}\left(G\left(0\right)H\left(0\right)\right)<1,$$

*where $\lambda_{max}\left(\cdot \right)$ denotes the maximum eigenvalue of a matrix with only real eigenvalues.*

### 2.2. Nonlinear Negative Imaginary Systems

In [12], negative imaginary systems theory has been recently generalized to nonlinear systems. Here, we review the main definitions and stability results of nonlinear negative imaginary systems. We consider the following multi-input multi-output (MIMO) general nonlinear system of the form

(5) $$\begin{array}{cc} \dot{x} & =f(x,u) \end{array}$$

(6) $$\begin{array}{cc} y & =h(x) \end{array}$$

where $f:R^{n}\times R^{m}\to R^{n}$ is Lipschitz continuous function and $h:R^{n}\to R^{m}$ is continuously differentiable function. The following definitions give a time-domain characterization of the nonlinear negative imaginary property of the nonlinear system (5) and (6).

**Definition** **3** ([12])**.** *The system *(5)* and *(6)* is said to be nonlinear negative imaginary if there exists a non-negative function $V:R^{n}\to R$ of a class $C^{1}$ such that the following dissipative inequality*

(7) $$\dot{V}\left(x\left(t\right)\right)\leq {\dot{y}}^{T}\left(t\right)u\left(t\right),$$

*holds for all t≥0. Here, the function V is called a storage function.*

Analogously to [22] for passive systems, we introduce a slightly stronger notions of the above definition for the purpose of stability analysis. We have the following two definitions.

**Definition** **4** ([12])**.** *The system *(5)* and *(6)* is said to be a marginally strictly nonlinear NI system if the dissipative inequality *(7)* is satisfied, and for all u and x such that*

(8) $$\dot{V}\left(x\right)={\dot{y}}^{T}\left(t\right)u\left(t\right)$$

*for all t>0, then $\lim_{t\to \infty }u\left(t\right)=c$, and c is a constant vector.*

**Definition** **5** ([12])**.** *The system *(5)* and *(6)* is said to be weakly strictly nonlinear NI system if it is marginally strictly nonlinear NI and globally asymptotically stable with u=0.*

**Remark** **1.** *For LTI systems of the form *(1)* and *(2)*, weakly strictly nonlinear negative imaginary property becomes strictly negative imaginary property. This can be readily seen by considering a positive definite storage function as $V\left(x\right)=\frac{1}{2}x^{T}Px$, where x is the state vector of the system and the matrix P is positive definite symmetric matrix which satisfies the LMI *(4)*. Differentiating V with respect to time, we have $\dot{V}\left(x\left(t\right)\right)={\dot{y}}^{T}u-{\tilde{y}}^{T}\tilde{y}$, where $\tilde{y}$ is the output of the auxiliary system given by $H\left(s\right)=sM\left(s\right)=LP{\left(sI-A\right)}^{-1}B-LPA^{-1}B$ which has no zeros on the imaginary axis except at the origin (see Lemma 3). Since the system is stable, u(t) can consist only of exponentially decaying terms and sinusoids (including zero frequency). Note that $\tilde{Y}\left(s\right)=H\left(s\right)U\left(s\right)$. The condition that H(jω) has no zeros on the imaginary axis implies it is not zero for all ω≠0, which guarantees the convergence of u(t) to a (possibly zero) constant.*

In what follows, we highlight the main robust stability result introduced in [12] for the positive feedback interconnection of two nonlinear NI systems. This nonlinear stability result will be used later to robustly stabilize the attitude of the quadrotor system.

**Figure 2:**
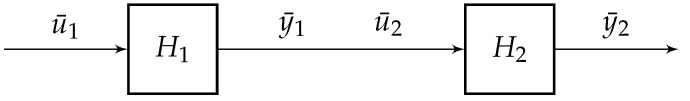
Open-loop interconnection of $H_{1}$ and $H_{2}$.

Now, consider the following general two MIMO nonlinear systems described by:

$$\begin{array}{cc} H_{1}:\;{\dot{x}}_{1} & =f_{1}\left(x_{1},u_{1}\right)\\ y_{1} & =h_{1}\left(x_{1}\right) \end{array}$$

and

$$\begin{array}{ccc} H_{2}: & \;{\dot{x}}_{2} & =f_{2}\left(x_{2},u_{2}\right)\\ & y_{2} & =h_{2}\left(x_{2}\right) \end{array}$$

where $h_{i}:R^{n}\to R^{n}$ is a $C^{1}$ function with $h_{i}\left(0\right)=0$, $f_{i}:R^{n}\times R^{n}\to R^{n}$ is continuous and locally Lipschitz in $x_{i}$ for bounded $u_{i}$, and where $f_{i}\left(0,0\right)=0$. We shall consider the open-loop interconnection of the systems $H_{1}$ and $H_{1}$ as shown in Figure 2. This interconnected system determines the stability properties of the closed-loop system, see [12]. We have the following assumptions for the open-loop interconnection of systems $H_{1}$ and $H_{2}$.

**Assumption** **1.** *For any constant ${\bar{u}}_{1}$, there exists a unique solution ${\bar{x}}_{1},{\bar{y}}_{1}$ to the equations*

$$\begin{array}{cc} 0 & =f_{1}\left({\bar{x}}_{1},{\bar{u}}_{1}\right)\\ {\bar{y}}_{1} & =h_{1}\left({\bar{x}}_{1}\right) \end{array}$$

*such that ${\bar{u}}_{1}\neq 0$ implies ${\bar{x}}_{1}\neq 0$ and the mapping ${\bar{u}}_{1}\mapsto {\bar{x}}_{1}$ is continuous.*

**Assumption** **2.** *For any constant ${\bar{u}}_{2}$, there exists a unique solution $\left({\bar{x}}_{2},{\bar{y}}_{2}\right)$ to the equations*

$$\begin{array}{cc} 0 & =f_{2}\left({\bar{x}}_{2},{\bar{u}}_{2}\right)\\ {\bar{y}}_{2} & =h_{2}\left({\bar{x}}_{2}\right) \end{array}$$

*such that ${\bar{u}}_{2}\neq 0$ implies ${\bar{x}}_{2}\neq 0$ and the mapping ${\bar{u}}_{2}\mapsto {\bar{x}}_{2}$ is continuous.*

**Assumption** **3.** *$h_{1}^{T}\left({\bar{x}}_{1}\right)h_{2}\left({\bar{x}}_{2}\right)\geq 0$, for any constant ${\bar{u}}_{1}$ where ${\bar{u}}_{2}={\bar{y}}_{1}$.*

**Assumption** **4.** *For any constant ${\bar{u}}_{1}$, let $\left({\bar{x}}_{1},{\bar{y}}_{1}\right)$ be defined as in Assumption 1 and $\left({\bar{x}}_{2},{\bar{y}}_{2}\right)$ be defined as in Assumption 2 where ${\bar{u}}_{2}={\bar{y}}_{1}$. Then there exits a constant 0<γ<1 such that for any ${\bar{u}}_{1}$ and with ${\bar{y}}_{2}$ defined as in Assumption 2, the following sector bound condition:*

(9) $${\bar{y}}_{2}^{T}{\bar{y}}_{2}\leq \gamma^{2}{\bar{u}}_{1}^{T}{\bar{u}}_{1},$$

*holds.*

The stability robustness of the positive feedback interconnections of systems $H_{1}$ and $H_{2}$ has been given in the next theorem using the Lyapunov theory and the LaSalle’s invariance principal.

**Theorem** **2** ([12])**.** *Consider a positive feedback interconnection of systems $H_{1}$ and $H_{2}$ where $u_{1}=y_{2}$, $u_{2}=y_{1}$. Suppose that the system $H_{1}$ is nonlinear NI and zero-state observable, and the system $H_{2}$ is weakly strictly nonlinear NI. Moreover, suppose that Assumptions 1–4 are satisfied. Then, the equilibrium point $\left(x_{1},x_{2}\right)=\left(0,0\right)$ of the closed-loop system of $H_{1}$ and $H_{2}$ is asymptotically stable.*

## 3. Quadrotor System

In this section, we aim to reveal the nonlinear negative imaginary structure of the quadrotor system using the Euler–Lagrange equation of the quadrotor. First we recall the kinematics and dynamics model of the quadrotor with parameters as shown in Table 1.

### 3.1. Kinematics Model

**Figure 3:**
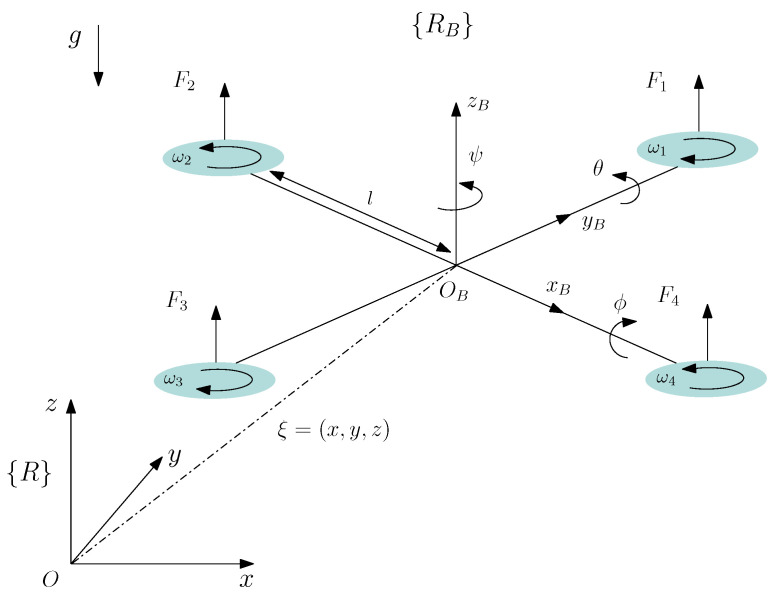
Quadrotor configuration with body-fixed frame and inertial frame.

Two reference frames are used to study the quadrotor system (see Figure 3): a reference frame fixed to the earth {R}(O,x,y,z), and a body-fixed frame $\left\{R_{B}\right\}\left\{O_{B},x_{B},y_{B},z_{B}\right\}$, where $O_{B}$ is fixed to the center of mass of the quadrotor. $\left\{R_{B}\right\}$ is related to {R} by a position vector $\xi ={\left[x,y,z\right]}^{T}$, describing the position of the center of gravity in $\left\{R_{B}\right\}$ relative to {R} and by a vector of three independent angles, known as Euler angles and denoted by $\eta ={\left[\phi ,\theta ,\psi \right]}^{T}$, which represent roll, pitch, and yaw angles of the quadrotor. It is assumed that the Euler angles are bounded as follows:

ϕ∈(−π/2,π/2),θ∈(−π/2,π/2),ψ∈(−π,π].

A vector in the body reference frame can be transformed into vectors in the earth reference frame. For example, given a force $F_{B}$, expressed using the coordinates of the body frame, the force F can be expressed in the coordinates of the earth frame as follows:

(10) $$F=R_{B\to E}F_{B}$$

where $R_{B\to E}$ is the transformation (rotation) matrix given by

(11) $$R:=\left[\begin{array}{ccc} C_{\theta }C_{\psi } & C_{\psi }S_{\theta }S_{\phi }-C_{\phi }S_{\psi } & C_{\phi }C_{\psi }S_{\theta }+S_{\phi }S_{\psi }\\ C_{\theta }S_{\psi } & S_{\theta }S_{\phi }S_{\psi }+C_{\phi }C_{\psi } & C_{\phi }S_{\theta }S_{\psi }-C_{\psi }S_{\phi }\\ -S_{\theta } & C_{\theta }S_{\phi } & C_{\theta }C_{\phi } \end{array}\right].$$

Here $S_{\left(\cdot \right)}$ and $C_{\left(\cdot \right)}$ represent the functions sin(·) and cos(·), respectively. Likewise, the relation between the angular velocity vector in the inertial frame $\omega ={\left[p,q,r\right]}^{T}$, and the angular velocity in the body frame $\eta ={\left[\phi ,\theta ,\psi \right]}^{T}$ is given as:

(12) $$\omega =W_{\eta }\dot{\eta }$$

where

(13) $$W_{\eta }:=\left[\begin{array}{ccc} 1 & 0 & -S_{\theta }\\ 0 & C_{\phi } & S_{\phi }C_{\theta }\\ 0 & -S_{\phi } & C_{\phi }C_{\theta } \end{array}\right].$$

**Remark** **2.** *For small-angle approximation, we obtain an equality between the Euler rates $\dot{\eta }={\left[\dot{\phi },\dot{\theta },\dot{\psi }\right]}^{T}$ and the angular velocity vector $\omega ={\left[p,q,r\right]}^{T}$, i.e., $W_{\eta }=I$.*

### 3.2. Euler–Lagrange Model: Nonlinear Negative Imaginary Structure

Here, an Euler–Lagrange approach is adopted in order to write the equations which describe the translational and rotational motion of the quadrotor. The Euler–Lagrange dynamics of the quadrotor are given by

(14) $$\frac{d}{dt}\left(\frac{\partial L}{\partial \dot{q}}\right)-\frac{\partial L}{\partial q}=F$$

where $q={\left[\xi^{T},\eta^{T}\right]}^{T}={\left[x,y,z,\phi ,\theta ,\psi \right]}^{T}$ is the generalized coordinates vector for the quadrotor, and $F={\left[F_{\xi }^{T},\tau^{T}\right]}^{T}$, is the input (generalized forces) of the system where $F_{\xi }$ is the thrust force and τ is total torque. The Lagrangian $L(q,\dot{q})$ of the quadrotor is the difference between the total kinetic energy *T* and the potential energy *P*; that is

(15) $$L=T_{trans}+T_{rot}-P.$$

Herein, $T_{trans}$ represents the translational kinetic energy, and is given by

(16) $$T_{trans}=\frac{1}{2}m{\dot{\xi }}^{T}\dot{\xi },$$

where *m* denotes the whole mass of the quadrotor. The term $T_{rot}$ represents the rotational kinetic energy, and is given by

(17) $$T_{rot}=\frac{1}{2}{\dot{\eta }}^{T}J\dot{\eta },$$

where J is positive-definite matrix denotes the rotational inertia matrix of the quadrotor and is defined in the body frame as

(18) $$J=\left[\begin{array}{ccc} J_{x} & 0 & 0\\ 0 & J_{y} & 0\\ 0 & 0 & J_{z} \end{array}\right].$$

The rotational inertia matrix J is diagonal due to the quadrotor’s symmetry with the four arms aligned with the body x− and y− axis. The potential energy is P=mgz, where *g* is the acceleration due to gravity. Then, the Lagrangian of a quadrotor is given as follows:

(19) $$L\left(q,\dot{q}\right)=\frac{1}{2}m{\dot{\xi }}^{T}\dot{\xi }+\frac{1}{2}{\dot{\eta }}^{T}J\dot{\eta }-mgz=\frac{1}{2}{\dot{q}}^{T}M\dot{q}+G\left(q\right),$$

where

(20) $$M=\left[\begin{array}{cc} mI_{3\times 3} & 0_{3\times 3}\\ 0_{3\times 3} & J \end{array}\right],\;G\left(q\right)={\left[0\;0\;\;-mg\;\;0\;\;0\;\;0\right]}^{T}.$$

To establish the nonlinear negative imaginary property of the quadrotor we express the equation (14) in terms of the generalized coordinates. Using the Lagrangian (19), Equation (14) can be written as follows

(21) $$M\left(q\right)\ddot{q}+C\left(q,\dot{q}\right)\dot{q}+G\left(q\right)=F,$$

where M(q) denotes inertia matrix (given in (20)) and is symmetric and positive definite. $C(q,\dot{q})$ is the Coriolis and centrifugal matrix where $C\left(q,\dot{q}\right)=\frac{d}{dt}M\left(q\right)-\frac{1}{2}\frac{\partial }{\partial q}\left({\dot{q}}^{T}M\right)$, the term G(q) is the gravitational vector where $G\left(q\right)=\frac{\partial P(q)}{\partial q}$, and F is the input of the quadrotor system. We assume that the matrix $C(q,\dot{q})$ is defined using the Christoffel symbols; then $\dot{M}-2C\left(q,\dot{q}\right)$ is skew-symmetric [23,24]. Furthermore, P(q) is assumed to have an absolute minimum at q=0.

In the following lemma, we show that the quadrotor system (21) with F as input and q as output is nonlinear negative imaginary.

**Lemma** **4.** *Consider the system *(21)* with input F and output q. Then the system *(21)* is nonlinear negative imaginary with a positive-definite storage function given by*

(22) $$V\left(q,\dot{q}\right)=\frac{1}{2}{\dot{q}}^{T}M\left(q\right)\dot{q}+P\left(q\right).$$

**Proof.**  It can be easily shown that *V* is positive definite since where M(q) is positive-definite matrix, and P(q) is a nonnegative scalar quantity. Taking the first time derivative of the function *V* we obtain

$$\begin{array}{cc} \frac{dV}{dt}\left(q,\dot{q}\right) & ={\dot{q}}^{T}M\left(q\right)\ddot{q}+\frac{1}{2}{\dot{q}}^{T}\dot{M}\left(q\right)\dot{q}+G\left(q\right)\dot{q}\\ \; & ={\dot{q}}^{T}\left[-C\left(q,\dot{q}\right)\dot{q}-G\left(q\right)+F\right]+\frac{1}{2}{\dot{q}}^{T}\dot{M}\left(q\right)\dot{q}+G\left(q\right)\dot{q}\\ \; & ={\dot{q}}^{T}F+\frac{1}{2}{\dot{q}}^{T}\left[\dot{M}\left(q\right)-2C\left(q,\dot{q}\right)\right]\dot{q}={\dot{q}}^{T}F, \end{array}$$

which shows that the systems is nonlinear negative system from F to q. □

**Remark** **3.** *By revealing the nonlinear negative imaginary structure of the quadrotor model, we can leverage powerful techniques from negative imaginary systems theory to be utilized in future work to achieve a broad range of control objectives for the quadrotor system.*

### 3.3. State-Space Model

Since the stability results of [12] deal with state-space representation of nonlinear systems, we aim here to use the state-space representation of the quadrotor rotational subsystem to design quadrotor attitude system, which is the main scope of this paper. We see that the Lagrangian contains no cross-terms in the kinetic energy combining $\dot{\xi }$ and $\dot{\eta }$, so the Euler–Lagrange Equation (14) partitions into two parts; that is, the translational and rotational components. The translational equation of the quadrotor is described by the following equation

(23) $$m\ddot{\xi }+\left(\begin{array}{c} 0\\ 0\\ mg \end{array}\right)=F_{\xi },$$

with $F_{\xi }$ is the thrust force generated by the four rotors and is given by $F_{\xi }=\sum_{i=1}^{4}b\omega_{i}^{2}$, where $\omega_{i}$ is *i*th-rotor’s speed and *b* is the thrust factor. The rotational subsystem describing the roll, pitch and yaw rotations of the quadrotor is described by the following equation

(24) $$J\left(\eta \right)\ddot{\eta }+\frac{d}{dt}\left\{J\left(\eta \right)\right\}\dot{\eta }-\frac{1}{2}\frac{\partial }{\partial \eta }\left({\dot{\eta }}^{T}J\left(\eta \right)\dot{\eta }\right)=\tau ,$$

or, by appropriate definition of variables,

(25) $$J\left(\eta \right)\ddot{\eta }+C\left(\eta ,\dot{\eta }\right)\dot{\eta }=\tau ,$$

where the input vector $\tau ={\left[\tau_{\phi },\tau_{\theta },\tau_{\psi }\right]}^{T}$ is the total torque in the pitch, roll, and yaw. The term $J\left(\eta \right)-2C\left(\eta ,\dot{\eta }\right)$ can be proved a skew-symmetric by using specific representation [24].

In matrix form, the vector τ is defined in terms of the four rotor speeds as follows,

(26) $$\tau =\left(\begin{array}{c} lb\left(\omega_{2}^{2}-\omega_{4}^{2}\right)\\ lb\left(\omega_{1}^{2}-\omega_{3}^{2}\right)\\ d\left(\omega_{1}^{2}+\omega_{3}^{2}-\omega_{2}^{2}-\omega_{4}^{2}\right) \end{array}\right),$$

where *l* is the arm length, the distance from the axis of rotation of the rotors to the center of the quadrotor, and *d* is the drag force. By defining the input of the quadrotor as follows

(27) $$\left(\begin{array}{c} F_{\xi }\\ \tau_{\phi }\\ \tau_{\theta }\\ \tau_{\psi } \end{array}\right)=\left(\begin{array}{c} u_{1}\\ u_{2}\\ u_{3}\\ u_{4} \end{array}\right)=\left(\begin{array}{cccc} b & b & b & b\\ 0 & b & 0 & -b\\ b & 0 & -b & 0\\ d & -d & d & -d \end{array}\right)\left(\begin{array}{c} \omega_{1}^{2}\\ \omega_{2}^{2}\\ \omega_{3}^{2}\\ \omega_{4}^{2} \end{array}\right),$$

we obtain the overall quadrotor dynamic model in the following form

(28) $$\begin{array}{cc} \ddot{x} & =-\left(\cos\phi \sin\theta \cos\psi +\sin\phi \sin\psi \right)\cdot \frac{u_{1}}{m}\\ \ddot{y} & =-\left(\cos\phi \sin\theta \sin\psi -\sin\phi \cos\psi \right)\cdot \frac{u_{1}}{m}\\ \ddot{z} & =g-\left(\cos\phi \cos\theta \right)\cdot \frac{u_{1}}{m}\\ \ddot{\phi } & =\dot{\theta }\dot{\psi }\left(\frac{J_{y}-J_{z}}{J_{x}}\right)-\frac{J_{r}}{J_{x}}\dot{\theta }g\left(u\right)+\frac{l}{J_{x}}u_{2}\\ \ddot{\theta } & =\dot{\phi }\dot{\psi }\left(\frac{J_{z}-I_{x}}{J_{y}}\right)+\frac{J_{r}}{J_{y}}\dot{\phi }g\left(u\right)+\frac{l}{J_{y}}u_{3}\\ \ddot{\psi } & =\dot{\phi }\dot{\theta }\left(\frac{J_{x}-J_{y}}{J_{z}}\right)+\frac{1}{J_{z}}u_{4} \end{array}$$

Introducing the abbreviation $g\left(u\right)=\omega_{1}-\omega_{2}+\omega_{3}-\omega_{4}$, the system (28) can be represented in the form $\dot{x}=f\left(x,u\right)$ with the 12-dimensional state vector $x={\left[x_{1},\ldots ,x_{12}\right]}^{T}={\left[\phi ,\dot{\phi },\theta ,\dot{\theta },\psi ,\dot{\psi },z,\dot{z},x,\dot{x},y,\dot{y}\right]}^{T}$, and the input vector $u={\left[u_{1},u_{2},u_{3},u_{4}\right]}^{T}$:

(29) $$f\left(x,u\right)=\left(\begin{array}{c} x_{2}\\ x_{4}x_{6}a_{1}-x_{4}a_{2}g\left(u\right)+b_{1}u_{2}\\ x_{4}\\ x_{2}x_{6}a_{3}+x_{2}a_{4}g\left(u\right)+b_{2}u_{3}\\ x_{6}\\ x_{4}x_{2}a_{5}+b_{3}u_{4}\\ x_{8}\\ g-\frac{u_{1}}{m}\cos x_{1}\cos x_{3}\\ x_{10}\\ -\frac{u_{1}}{m}\left(\sin x_{1}\sin x_{5}+\cos x_{1}\sin x_{3}\cos x_{5}\right)\\ x_{12}\\ \frac{u_{1}}{m}\left(\sin x_{1}\cos x_{5}-\cos x_{1}\sin x_{3}\sin x_{5}\right) \end{array}\right)$$

where

$$\begin{array}{cc} a_{1}=\left(J_{y}-J_{z}\right)/J_{x} & b_{1}=l/J_{x}\\ a_{2}=J_{r}/J_{x} & b_{2}=l/J_{y}\\ a_{3}=\left(J_{z}-J_{x}\right)/J_{y} & b_{3}=1/J_{z}\\ a_{4}=J_{r}/J_{y}\\ a_{5}=\left(J_{x}-J_{y}\right)/J_{z} \end{array}$$

## 4. Attitude Control Design

As it can be seen from the previous section, the state-space model of the quadrotor can be divided into two subsystems, one of which, the rotational subsystem, describes the dynamics of the attitude (i.e., the angles) and the other describes the translation of the quadrotor. In this paper, we are interested in the problem of stabilizing the attitude of the quadrotor around a desired reference signal by using a nonlinear negative imaginary approach. For this purpose we confine ourselves to the rotational subsystem whose state is a restriction to the last 6 components of x representing the roll, pitch and yaw angles and their time derivatives. The rotational subsystem is then described by the differential equation

(30) $$\dot{x}=f_{\alpha }\left(x,u\right)=\left(\begin{array}{c} x_{2}\\ x_{4}x_{6}a_{1}-x_{4}a_{2}g\left(u\right)+b_{1}u_{2}\\ x_{4}\\ x_{2}x_{6}a_{3}+x_{2}a_{4}g\left(u\right)+b_{2}u_{3}\\ x_{6}\\ x_{4}x_{2}a_{5}+b_{3}u_{4} \end{array}\right).$$

In recent studies, the use of a multi-loop control architecture has been proposed for a variety of quadrotor control problems; see for instance [25,26]. In this section, we propose an inner-outer loop architecture based on the nonlinear negative theory [12] to robustly stabilize the attitude, i.e., the Euler angles, around desired reference signal $\eta_{d}={\left[\phi_{d},\theta_{d},\psi_{d}\right]}^{T}={\left[x_{1}^{d},x_{3}^{d},x_{5}^{d}\right]}^{T}$, where the angular velocity measurements are not needed.

### 4.1. Inner-Control Loop

**Figure 4:**
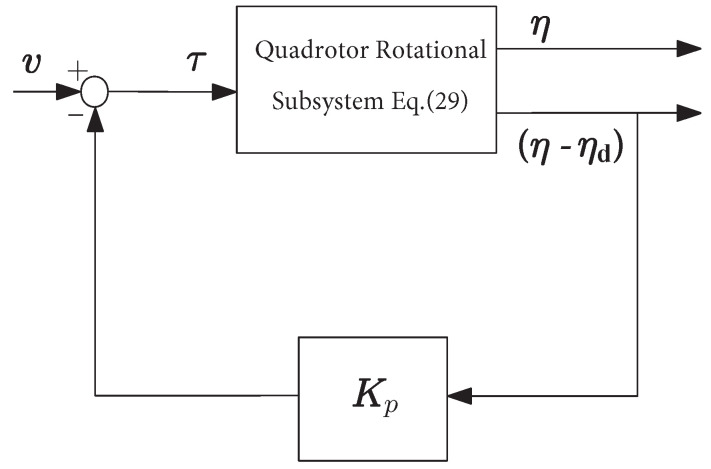
Inner-control loop.

The inner-control loop is mainly designed due to the free motion behavior of the quadrotor. We define the following feedback control law:

(31) $$\tau =-K_{p}\left(\eta -\eta_{d}\right)+v$$

where $v={\left[v_{1},v_{2},v_{3}\right]}^{T}$ denotes the new input torque of the quadrotor, and $K_{p}=\mathrm{diag}\left(k_{p}^{\phi },k_{p}^{\theta },k_{p}^{\psi }\right)$ is a positive diagonal matrix and the diagonal elements are used as tuning parameters. The architecture of the inner-loop can be interpreted as a proportional-only controller as seen in Figure 4 below.

The designed torque τ is then defined as follows,

(32) $$\tau =\left(\begin{array}{c} u_{2}\\ u_{3}\\ u_{4} \end{array}\right)=\left(\begin{array}{c} -k_{p}^{\phi }\left(x_{1}-x_{1}^{d}\right)+v_{1}\\ -k_{p}^{\theta }\left(x_{3}-x_{3}^{d}\right)+v_{2}\\ -k_{p}^{\psi }\left(x_{5}-x_{5}^{d}\right)+v_{3} \end{array}\right)$$

Using (32), and setting the desired reference signal $\eta_{d}={\left[x_{1}^{d},x_{3}^{d},x_{5}^{d}\right]}^{T}=0$, the rotational subsystem can be put in the form $\dot{x}={\tilde{f}}_{\alpha }\left(x,u\right)$:

$${\tilde{f}}_{\alpha }\left(x,u\right)=\left(\begin{array}{c} x_{2}\\ x_{4}x_{6}a_{1}-x_{4}a_{2}g\left(u\right)-b_{1}k_{p}^{\phi }\left(x_{1}-x_{1}^{d}\right)+b_{1}v_{1}\\ x_{4}\\ x_{2}x_{6}a_{3}+x_{2}a_{4}g\left(u\right)-b_{2}k_{p}^{\theta }\left(x_{2}-x_{3}^{d}\right)+b_{2}v_{2}\\ x_{6}\\ x_{4}x_{2}a_{5}-b_{3}k_{p}^{\psi }\left(x_{3}-x_{3}^{d}\right)+b_{3}v_{3} \end{array}\right),$$

or equivalently,

(33) $$J\left(\eta \right)\ddot{\eta }+C\left(\eta ,\dot{\eta }\right)\dot{\eta }=-K_{p}\eta +v.$$

The above rotational dynamical system (33) can be seen as a nonlinear negative imaginary system from the input v to the output η according to the following lemma.

**Lemma** **5.** *Consider the quadrotor rotational subsystem *(33)* with v as input and η as output. Then, the system *(33)* is a nonlinear negative imaginary system with respect to the following positive-definite storage function,*

(34) $$V\left(\eta ,\dot{\eta }\right)=\frac{1}{2}{\dot{\eta }}^{T}J\left(\eta \right)\dot{\eta }+\frac{1}{2}\eta^{T}K_{p}\eta .$$

**Proof.**  It can be easily shown that *V* is a valid storage function; since the rotational inertia matrix J is positive definite and $K_{p}>0$. Taking the time derivative of *V* we obtain

$$\begin{array}{cc} \frac{dV}{dt}\left(\eta ,\dot{\eta }\right) & ={\dot{\eta }}^{T}J\left(\eta \right)\ddot{\eta }+\frac{1}{2}{\dot{\eta }}^{T}\dot{J}\left(\eta \right)\dot{\eta }+\eta^{T}K_{p}\dot{\eta }\\ \; & ={\dot{\eta }}^{T}\left[-C\left(\eta ,\dot{\eta }\right)\dot{\eta }-K_{p}\eta +v\right]+\frac{1}{2}{\dot{\eta }}^{T}\dot{J}\left(\eta \right)\dot{\eta }+\eta^{T}K_{p}\dot{\eta }\\ \; & ={\dot{\eta }}^{T}v+\frac{1}{2}{\dot{\eta }}^{T}\left[\dot{J}\left(\eta \right)-2C\left(\eta ,\dot{\eta }\right)\right]\dot{\eta }={\dot{\eta }}^{T}v, \end{array}$$

which shows that the system (33) is nonlinear negative imaginary from the input v to the output η. □

### 4.2. Outer-Control Loop

We aim here to design the outer control loop of the rotational subsystem in order to get a positive-feedback closed-loop system which guarantees the asymptotic stability of the quadrotor attitude in view of Theorem 2. For simplicity, we will use the following linear MIMO integral resonant controller as the outer control loop controller,

(35) $$C_{v}\left(s\right)={\left[sI+\Gamma \Delta \right]}^{-1}\Gamma .$$

Here, Γ and Δ are positive-definite matrices given by $\Delta =\mathrm{diag}\left(\delta ,\delta ,\delta \right)$, and $\Gamma =\mathrm{diag}\left(\Gamma ,\Gamma ,\Gamma \right)$, where δ and Γ are tuning parameters. The transfer function matrix $C_{v}\left(s\right)$ is strictly negative imaginary [16]. The dc-gain (the gain of the system at steady-state) of the controller is $C_{v}\left(0\right)=\Delta^{-1}$.

**Figure 5:**
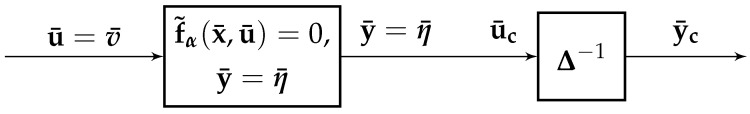
Open-loop interconnection (in the steady-state case) of the quadrotor rotational subsystem (33) and the SNI controller (35) (where ‘*c*’ refers to the controller).

In order to apply Theorem 2, we need to validate Assumptions 1–4 on the open-loop interconnection (as shown in Figure 5) of the quadrotor rotational subsystem (33) and the SNI controller (35) in the steady-state case. We have ${\tilde{f}}_{\alpha }\left(\bar{x},\bar{u}\right)=0$, this implies ${\bar{x}}_{2}={\bar{x}}_{4}={\bar{x}}_{6}=0$ and

(36) $$\begin{array}{cc} {\bar{x}}_{1} & =\frac{{\bar{v}}_{1}}{k_{p}^{\phi }},\\ {\bar{x}}_{3} & =\frac{{\bar{v}}_{2}}{k_{p}^{\theta }},\\ {\bar{x}}_{5} & =\frac{{\bar{v}}_{3}}{k_{p}^{\psi }}, \end{array}$$

which shows for every constant value of $\bar{v}$ there is a corresponding unique value of $\bar{x}$ and hence Assumption 1 holds. Furthermore, Assumption 2 trivially holds since the controller (35) is a linear system.

We can easily see that Assumption 3 is valid since we have ${\bar{y}}_{c}=\Delta^{-1}{\bar{u}}_{c}=\frac{1}{\delta }\bar{y}$, where δ>0, it yields

$${\bar{y}}^{T}{\bar{y}}_{c}=\frac{1}{\delta }{\bar{y}}^{T}\bar{y}\geq 0.$$

Lastly, using (36) we see that

$$\begin{array}{cc} {\bar{y}}_{c}^{T}{\bar{y}}_{c} & =\frac{1}{\delta^{2}}\left[{\bar{x}}_{1}+{\bar{x}}_{3}+{\bar{x}}_{5}\right]\\ & =\frac{1}{\delta^{2}}\left[\frac{{\bar{v}}_{1}^{2}}{{\left(k_{p}^{\phi }\right)}^{2}}+\frac{{\bar{v}}_{2}^{2}}{{\left(k_{p}^{\theta }\right)}^{2}}+\frac{{\bar{v}}_{3}^{2}}{{\left(k_{p}^{\psi }\right)}^{2}}\right]\\ \; & \leq \frac{1}{\delta^{2}}\max_{i=\phi ,\theta ,\psi }\left\{\frac{1}{{\left(k_{p}^{i}\right)}^{2}}\right\}\left({\bar{v}}_{1}^{2}+{\bar{v}}_{2}^{2}+{\bar{v}}_{3}^{2}\right)\\ \; & =\frac{1}{\delta^{2}}\max_{i=\phi ,\theta ,\psi }\left\{\frac{1}{{\left(k_{p}^{i}\right)}^{2}}\right\}{\bar{v}}^{T}\bar{v}\\ \; & =\gamma^{2}{\bar{v}}^{T}\bar{v}, \end{array}$$

where

$$\gamma^{2}=\frac{1}{\delta^{2}}\max_{i=\phi ,\theta ,\psi }\left\{\frac{1}{{\left(k_{p}^{i}\right)}^{2}}\right\}.$$

Thus, to ensure that Assumption 4 is valid such that $\gamma^{2}<1$, we choose the tuning parameter δ such that:

(37) $$\delta^{2}>\max_{i=\phi ,\theta ,\psi }\left\{\frac{1}{{\left(k_{p}^{i}\right)}^{2}}\right\}.$$

Based upon the above arguments, we have found a lower bound on the DC-gain of the outer controller that is necessary to achieve asymptotic stability of the attitude vector around the reference signal by virtue of Theorem 2. We summarize the above result in the following theorem.

**Figure 6:**
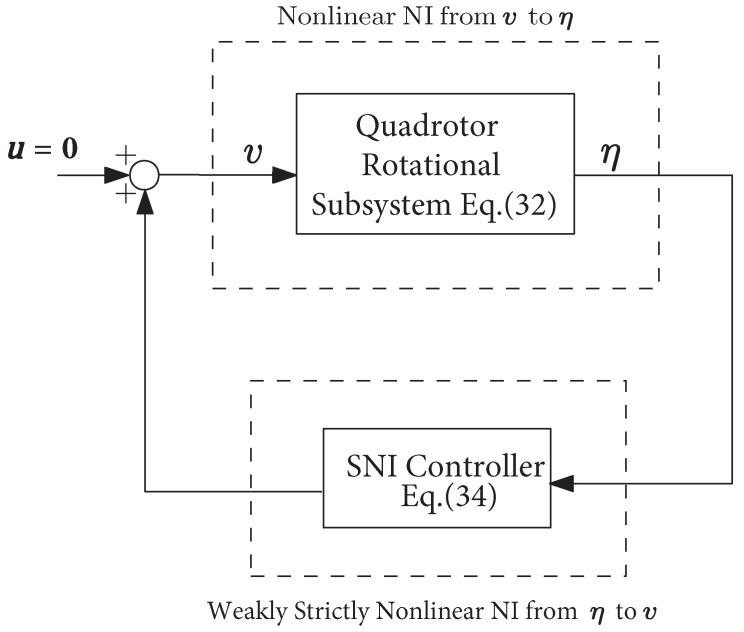
Block diagram of the proposed attitude control system comprised of a positive feedback interconnection of the rotational subsystem (33) and SNI controller (35).

**Theorem** **3.** *Consider the closed-loop system, as in Figure 6, of the quadrotor rotational subsystem *(33)* and the strictly negative imaginary controller *(35)*. Assume that the system *(33)* is zero-state observable, and the condition *(37)* is satisfied. Then the closed-loop system is asymptotically stable.*

**Proof.**  The proof follows from the proof of Theorem 2 along with remark 1. □

**Remark** **4.** *The above stability result leads to a robust control system since the stability is guaranteed irrespective of the quadrotor and controller parameters so long as the condition *(37)* is satisfied.*

## 5. Simulation Results

**Figure 7:**
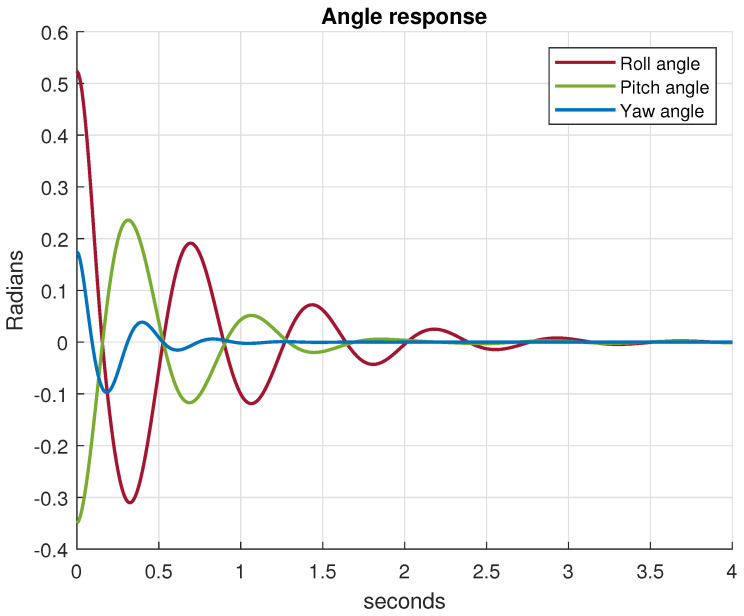
Euler angles during attitude control.

In order to verify the proposed attitude control method in this paper, we present a simulation results of the underlying quadrotor system tracking a desired reference attitude $\eta_{d}$. The quadrotor parameters used in the simulation are given as follows: $m=0.5\;\mathrm{kg},\;I_{xx}=I_{yy}=4.85\times 10^{-3}\;\mathrm{kg}\cdot m^{2},\;I_{zz}=8.81\times 10^{-3}\;\mathrm{kg}\cdot m^{2},\;g=9.81\;m/s^{2},$ $b=2.92\times 10^{-6}\;{\mathrm{Ns}}^{2},\;d=1.12\times 10^{-7}\;{\mathrm{Nms}}^{2}$. Using these parameters, the quadrotor rotational subsystem (33) is then implemented in MATLAB/Simulink for a simulation. The parameters of the inner-loop controller are chosen as $k_{p}^{\phi }=k_{p}^{\theta }=k_{p}^{\psi }=5$. Based on the result of Theorem 3, the tuning gains of the outer controller are set as $\Delta =\mathrm{diag}\left(0.3,0.3,0.3\right)$, and $\Gamma =\mathrm{diag}\left(160,160,160\right)$. We assume that the initial state of the attitude vector is ${\left(30,-20,10\right)}^{T}$, where the control goal is to stabilize the quadrotor at a hovering position, i.e., $\eta_{d}=0$. The obtained control result is shown in Figure 7 as a time plot of all Euler angles of the quadrotor system. The simulations show that the proposed control method asymptotically stabilize the attitude of the quadrotor to the desired reference signal.

## 6. Conclusions and Future Directions

The nonlinear negative imaginary systems theory has been employed to control the attitude of the quadrotor model based on the nonlinear NI property of the quadrotor rotational subsystem. The designed attitude control doesn’t rely on the angular velocity measurements through an appropriate design. In specific, an inner-outer loop architecture has been proposed to design a positive feedback control system that robustly stabilizes the quadrotor’s attitude in the face of model uncertainties and disturbances. Future direction of the proposed nonlinear negative imaginary approach is to address other quadrotor control problems (trajectory tracking, altitude control, etc.) and then complement the control applied to the quadrotor.

## Figures and Tables

**Table 1:** Quadrotor Parameters.

Definition	Parameter	Unit
Quadrotor mass	*m*	kg
Gravitational acceleration	*g*	$m/s^{2}$
Arm length	*l*	m
Thrust coefficient	*b*	$N\cdot s^{2}/{\mathrm{rad}}^{2}$
Drag coefficient	*d*	$N\cdot s^{2}/{\mathrm{rad}}^{2}$
Roll inertia x-axis	$J_{x}$	$\mathrm{kg}\cdot m^{2}$
Pitch inertia y-axis	$J_{y}$	$\mathrm{kg}\cdot m^{2}$
Yaw inertia	$J_{z}$	$\mathrm{kg}\cdot m^{2}$
Rotational Inertial	$J_{R}$	$\mathrm{kg}\cdot m^{2}$

## Data Availability

Not applicable.
